# Draft Genome Sequence of a *vanA*-Type Vancomycin-Resistant Reference Strain, *Enterococcus faecium* ATCC 51559

**DOI:** 10.1128/genomeA.01053-15

**Published:** 2015-09-10

**Authors:** Kidon Sung, Saeed Khan, Bernard Marasa, Seonggi Min, Ohgew Kweon, Nawaz Mohamed, Carl Cerniglia

**Affiliations:** a Division of Microbiology, National Center for Toxicological Research (NCTR), U.S. Food and Drug Administration, Jefferson, Arkansas, USA; b Office of Scientific Coordination, National Center for Toxicological Research (NCTR), U.S. Food and Drug Administration, Jefferson, Arkansas, USA

## Abstract

Vancomycin-resistant *Enterococcus faecium* has emerged as a multidrug-resistant pathogen in hospital settings. Here, we present the draft genome sequence of a high-level vancomycin-resistant strain, *E. faecium* ATCC 51559, which is employed as a standard laboratory *vanA* genotype-positive control strain for clinical and laboratory studies.

## GENOME ANNOUNCEMENT

Vancomycin-resistant enterococci (VRE) cause one-third of the most common bloodstream infections among hospitalized patients (1, 2). VRE isolates that exhibit a high level of resistance to vancomycin carry the *vanA* gene, which is present on a 10.8-kb transposon, Tn*1546* (3). This mobile genetic element can transfer to other pathogenic microorganisms and make them resistant to vancomycin. Since vancomycin is considered one of the last-resort antibiotics to treat infections caused by Gram-positive bacteria, management of VRE infections becomes difficult. Accurate and prompt detection of the antimicrobial susceptibility profiles of VREs is therefore necessary to prevent and control nosocomial outbreaks, allowing patients to be administered appropriate drugs. Several studies of the composition of plating/broth medium and growth conditions have been conducted to validate accuracy of (4, 5) and influence on antibiotic susceptibility profiles of VREs (6, 7). The effect of plating medium, however, was not correlated with the presence of vancomycin resistance genes in these studies. *E. faecium* ATCC 51599, a clinical isolate that was isolated from a patient in Brooklyn, New York, is used as a quality control strain for antimicrobial susceptibility testing (8). It contains the *vanA* gene on transposon Tn*1546*, which is quite prone to mutations and proved very helpful in understanding the phenomenon of vancomycin heteroresistance in different growth media (9, 10).

The genomic DNA of *E. faecium* 51599 was extracted using a Master Pure Gram-positive DNA purification kit (Epicentre Biotechnologies). A TruSeq DNA library preparation kit (Illumina) and TruSeq paired-end cluster kit were used for the DNA preparation and cluster generation for sequencing on a HiSeq system, respectively. An Illumina HiSeq 2500 system was used for sequencing. *De novo* assembly of a total of 37,959,664 high-quality paired-end reads (100 bp in length) was conducted using the CLC genomics workbench 6.5.1 (CLC Bio), and further genome annotation was performed using an annotation method GeneMarkS+ in the NCBI Prokaryotic Genome Annotation Pipeline (http://www.ncbi.nlm.nih.gov/genomes/static/Pipeline.html). The draft genome sequence of *E. faecium* 51599 was 2,953,574 bp in length with a G+C content of 37.7%, which is distributed in 160 contigs (*N*_50_ length, 52,737; average coverage, 2155.0×) with 2,831 coding sequences (CDS) and 56 RNAs.

### Nucleotide sequence accession numbers.

This whole-genome shotgun project has been deposited at DDBJ/EMBL/GenBank under the accession number JSVT00000000. The version described in this paper is version JSVT00000000.1.
